# Protein sequence information extraction and subcellular localization prediction with gapped *k*-Mer method

**DOI:** 10.1186/s12859-019-3232-4

**Published:** 2019-12-30

**Authors:** Yu-hua Yao, Ya-ping Lv, Ling Li, Hui-min Xu, Bin-bin Ji, Jing Chen, Chun Li, Bo Liao, Xu-ying Nan

**Affiliations:** 1 School of Mathematics and Statistics, Hainan Normal University, Haikou, 571158 China; 2 College of Life Sciences, Zhejiang Sci-Tech University, Hangzhou, 310018 China; 3 Basic Courses Department, Zhejiang Shuren University, Hangzhou, 310015 China; 4 School of Chemistry and Chemical Engineering, Hainan Normal University, Haikou, 571158 China

**Keywords:** Physicochemical properties, Position-specific score matrix, Gene ontology, Principal component analysis, Support vector machine

## Abstract

**Background:**

Subcellular localization prediction of protein is an important component of bioinformatics, which has great importance for drug design and other applications. A multitude of computational tools for proteins subcellular location have been developed in the recent decades, however, existing methods differ in the protein sequence representation techniques and classification algorithms adopted.

**Results:**

In this paper, we firstly introduce two kinds of protein sequences encoding schemes: dipeptide information with space and Gapped k-mer information. Then, the Gapped k-mer calculation method which is based on quad-tree is also introduced.

**Conclusions:**

>From the prediction results, this method not only reduces the dimension, but also improves the prediction precision of protein subcellular localization.

## Background

Proteins must be in a particular area in the cell for the participation in normal life activities (such as the mitochondria, nucleus, cytoplasm, etc). However, a multitude of protein sequences are increasingly identified into public biology databanks in post genome era, which results from the development of high-throughput technology. Therefore, it is indispensable to develop an automated method for fast and accurately annotating the subcellular attributes proteins. Predicting the subcellular locations of proteins can provide useful hints about the function of proteins, increase our understanding of the mechanisms of certain diseases, and ultimately help develop new drugs [1]. Such, more feature information representing the protein sequence should be extracted [2–5].

Apoptosis refers to the orderly death of genetically controlled cells in order to maintain internal environmental stability. Apoptosis is not only a special cell death type, but also has important biological significance and complex molecular biology mechanism [6, 7]. Therefore, the subcellular location of the apoptosis protein is a key step in understanding its working mechanism. However, traditional experimental methods for predicting the location of apoptosis proteins seem to be time-consuming and laborious [8]. In 2003, Zhou and Doctor [9] firstly put forward subcellular location of apoptosis proteins. Based on their research, many prediction algorithms are proposed one after another, including PseAAC with FKNN, PseAAC with SVM, distance frequency with SVM, covariance transformation, deep learning, fusion methods [9–22]. And GO annotation [23, 24], discrete wavelet transform [25, 26] and other methods were also introduced. Another key step in protein subcellular location prediction is classification algorithms. At present, support vector machine [27, 28], Bayesian [29], hidden Markov model [30], K-nearest neighbor, neural network [31] and others were widely used.

The amino acid composition of protein sequence is the simplest sequence statistics. However, AAC only take the information of the whole protein sequences into consideration, and ignore the partial order information. Therefore, some scholars consider the dipeptide composition information, and someone also proposed a prediction method which is based on *k*-peptide information. Compared to AAC, *k*-peptide information could be get a better predict results. But when *k* is larger, there exist dimension disaster, bad effects and some other problems. When doing the homologous sequence alignment, we will consider space penalty. By this, the best match between two sequences will be found. When extracting *k*-peptide information of sequences, we also take *k*-peptide information extraction with the space into consideration. The detailed description of the novel representation will be shown in the following section.

## Materials and methods

### Datasets

We first use two benchmark datasets constructed by the previous investigators. The ZD98 dataset, constructed by Zhou and Doctor [32], which has 43 cytoplasmic proteins, 30 plasma membrane-bound proteins, 13 mitochondrial proteins and 12 other proteins. The second dataset, CL317 [33], are divided into six subcellular locations with 112 cytoplasmic proteins, 55 membrane proteins, 34 mitochondrial proteins, 17 secreted proteins, 52 nuclear proteins and 47 endoplasmic reticulum proteins. Proteins in these two datasets are extracted from Swiss-Prot database. Although two datasets have small size, they are widely used in previous studies. Meanwhile, in order to test the effectiveness of the methods in large datasets, we also select Gram-negative (Gneg) dataset, which contains 1456 sequences in eight subcellular. The detailed information is listed in Table 1.

**Table 1:** The composition of Gneg 1456 dataset

Subcellular location	Number of proteins
Cell inner membrane	557
Cell outer membrane	124
Cytoplasm	410
Extracellular	133
Fimbrium	32
Flagellum	32
Nucleoid	8
Periplasm	180
Sum	1456

### Feature extraction

**Dipeptide information with the blank space** Before we start to discuss peptide information with spaces, we first study the simple dipeptide information with spaces. The basic form is “XDY”, where X and Y are one of amino acids, D is a certain number of spaces. D is reduced to *d*, 0 ≤ *d* ≤ *l* − 2. The *l* is the length of protein sequence. It indicates the common dipeptide information when *d* = 0. Obviously, the *d* value will affect the results of prediction directly.

### Gapped *k*-mer

Peptide information extraction method without spaces was abbreviated as *k*-mer and with spaces method was abbreviated as Gapped *k*-mer. In brief, with a space instead of some amino acids in dipeptide information method, then statistics all the different types of the peptides frequency.

If statistics *k*-peptide information of 20 kinds of amino acids directly, then it will get 20^*k*^ and 21^*k*^ dimension vector for *k*-mer and Gapped *k*-mer, respectively. The dimension of feature vector is very high when *k* > 3. This case will result in time-consuming classification forecast operation and cause too much redundant information. So in order to reduce the dimension, we adopted the following two kinds of measures:

1. In statistical *k*-peptide information, we only consider that kind of *k*-peptide, which was used in the training dataset, instead of all the different types of *k*-peptide. If we take all kinds of *k*-peptide, it will produce many zero vectors which have no effect on classification and cause the large dimension. Take the ZD98 dataset as test, if we consider all of the different 4-peptide when *k* = 4, *k*-mer will get 20^4^ =160,000 dimension feature vector. If only statistical all 4-peptide of ZD98 datasets, it will produce 22,265-dimensional feature vector. Compared to the former, dimension was reduced by 86.09%. Though dimension is still very big, it reduces the dimension feature vector in a certain extent. What’s more, the larger the *k*, the greater the proportion of reduced.
2. The amino acid reduced. 20 kinds of amino acids in a certain way were reduced into a few classes, which can greatly reduce the feature vector dimensions. Here we use a reduced solution which was used by a lot of researchers. According to the physical and chemical properties of amino acids, 20 kinds of amino acid were reduced to classify. Table 2 presented a detailed reduced scheme.

**Table 2:** Reduced scheme of amino acid

Classification	Shorthand	Abbreviation
Hydrophilic	L	R, D, E, N, Q, K, H
Hydrophobic	B	L, I, V, A, M, F
neutral	W	S, T, Y, W
proline	P	P
glycine	G	G
cysteine	C	C

Through these two measures, when extracting characteristic information with *k*-mer and Gapped *k*-mer, the dimensions of the feature vector will be greatly reduced. Such as extracting *k*-mer characteristic information with *k*-mer in ZD98 dataset, the dimension has been reduced to 1071. The dimension was reduced by 99.33%.

The *k*-mer was used to statistic *k*-frequency of protein sequence. For example, a protein sequence *S* can be represented as follows:

1 $$ {V}_k(S)={\left[{v}_1,{v}_2,...,{v}_i,...,{v}_n\right]}^T $$

where *n* is the varity of *k*-peptide, *v*_*i*_(*i* = 1, 2, ..., *n*) is the frequency of *i*-th *k*-peptide in protein sequence S.

The meaning of Gapped *k*-mer is statistics frequency with space *k*-peptide in the protein sequence S:

2 $$ {V}_{k,g}(S)={\left[{v}_1,{v}_2,...,{v}_i,...,{v}_m\right]}^T $$

where *m* is the varity of *k*-peptide with g spaces, *v*_*i*_(*i* = 1, 2, ..., *m*) is the frequency of *i*-th *k*-peptide with g spaces in protein sequence S.

Such as, a piece of protein sequence “RIAVYYPG”. According to the reduced solution of Table 1, the sequence can be reduced to “LBBBWWG”. The types of 3-mer statistics are “LBB, BBB, BBW, BWW, WWG”. There are five types and a protein can be represented by a 5-dimension vector with *k*-mer method. The number of spaces will be considered by Gapped 3-mer. If the number of spaces is 1, then the types of Gapped 3-mer are “_BB, _BW, _WW, _WG, L_B, B_W, W_G, B_B, LB_, BB_, BW_, WW_”. There are 12 kinds of types. Thus, a 12-dimension vector can be obtained by Gapped *k*-mer.

Here we should note that the “BBB” and “BBW” of 3-mer statistics in the above example will be regarded as belonging to the same type “BB_”. *k*-mer can be thought of a special case for Gapped *k*-mer (that is, the space is zero).

For a long enough protein sequence, according to some kind of reduced solution, the sequence can be reduced into *t* classes. So the dimension of feature vector is *t*^*k*^ for *k*-mer method. However, the dimension of feature vector is ${C}_k^g{t}^{k-\mathrm{g}}$ for Gapped *k*-mer method (the number of space is *g*). In general, when *k* is small, feature vector dimensions of Gapped *k*-mer is lower than *k*-mer.

In the classified prediction model of training and testing, we need to consider selection problem of parameters *k* and spaces (*g*).

### Gapped *k*-mer calculation method based on multi-tree

Here we first introduce a simple Gapped *k*-mer calculation method, which is based on multi-tree. For a scheme reducing amino acids into n classes, *k*-mer statistics of *k* peptide can be regarded as the kinds of statistics, i.e. a full depth *k* of *n* types leaf nodes in the tree. The first amino acid of n peptide is the head node, the second is the second straton node and so on. Finally the *k* layer is the leaf node. By comparing all k peptide in protein sequence to traverse the full *n* fork tree, and accumulating number of occurrences corresponding to a leaf node at the same time. Finally, the frequency of the leaf node corresponds to frequency of *k*-peptide. For Gapped *k*-mer, we need to consider the location of the space appears. If spaces appear in the m location of k peptide, the m layer of the original *k* peptide would be removed. Then merging the lower subtree, summing the frequency of the corresponding leaf node, and the frequency of gapped *k*-mer is obtained. It’s similar for the multiple spaces. The following is a simple example.

**Fig. 1:**
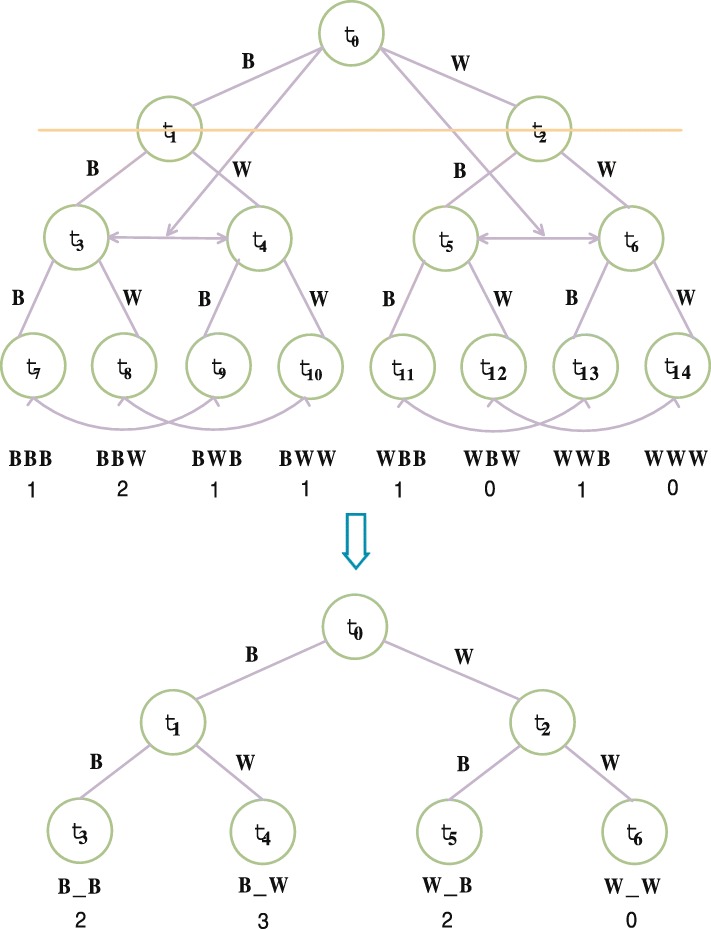
Gapped *k*-mer calculation method based on multi-treeand the final binary tree with 1 space in Gapped *k*-mer

A short sequence which has been reduced, Seq = BBBWWBBWB. Through the statistics of binary tree 3 peptide, frequency is shown in Fig. 1.

**Fig. 2:**
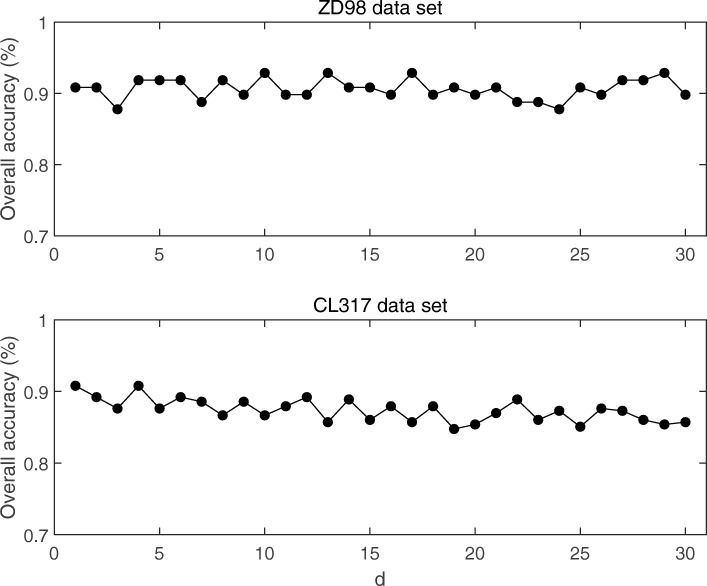
ZD98 and CL317 classification results with the change of *d*

Statistics the frequency of type 3-peptides with 1 space in Gapped *k*-mer. If the second position is space, such as the 3-peptides frequency of “X_Y” type. At this time you only need to delete the second floor. Merging its lower subtree, as shown in the Fig. 2, the final binary tree is the below in Fig. 2.

The frequency of all three peptides, such as “X_Y” is obtained by this method. The frequency of “B_B” types of the peptides is 2.

When using the same method to statistic form, such as “_XY”, the frequency of all 3 peptides, we just eliminating the head node, and merging the lower two subtrees.

Through the tree structure, we only need to construct the *k*-mer multi-tree. Then we can get all Gapped *k*-mer characteristic information vector, which is a simple statistical method.

### Support vector machine

Among these classifier, SVM exhibits quite promising results [34]. Support vector machine (SVM), introduced by Vapnik [35], was a kernel-based learning algorithm based on statistical learning theory. Protein subcellular location prediction is usually formulated as a multi-class classification problem, which is commonly solved by a decomposing and reconstructing procedure when the binary class SVM is implied. There are several methods to extend the SVM for classifying multi-class problems, for example ‘One-Versus-Rest (OVR)’ [36], ‘One-Versus-One (OVO)’ [37]. The latter is adopted in this paper. For a *k*-classification problem, k*(*k*-1)/2 classifiers need to be constructed. Meanwhile, the radial basis function (RBF) was selected as the kernel function due to that it outperforms the other kinds of kernel functions. Then a simple grid search strategy over *C* and γ values based on 10-fold cross-validation for each dataset was selected, where *C* and γ were allowed to take the values only between 2^−5^ to 2^5^.

### Evaluation methods

As is well known, independent dataset test, K-fold (such as five-fold or ten-fold) cross-validation (subsampling test), and jackknife test (leave-one-out cross validation (LOOCV)) are often used to examine a predictor’s effectiveness in practical application. Because considerable arbitrariness exists in the independent dataset test and K-fold cross-validation, and only the jackknife test is the least arbitrary that can always yield a unique result for a given benchmark dataset. Accordingly, the jackknife test is adopted here to examine the quality of the present predictor. For comprehensive evaluation, sensitivity (Sn), specificity (Sp) and Matthew’s correlation coefficient (MCC), as well as the overall accuracy (OA) over the entire dataset are reported. These parameters were detailed in the following equations:

3 $$ \mathrm{Specificity}=\frac{TN}{TN+ FP} $$

4 $$ \mathrm{Sensitivity}=\frac{TP}{TP+ FN} $$

5 $$ OA=\frac{\sum \limits_{i=1}^kT{P}_i}{N} $$

6 $$ {\displaystyle \begin{array}{l} MCC=\\ {}\frac{TP\times TN- FP\times FN}{\sqrt{\left( TP+ FN\right)\times \left( TP+ FP\right)\times \left( TN+ FN\right)\times \left( TN+ FP\right)}}\end{array}} $$

where *TP*, *TN*, *FP* and *FN* were the number of true positives, true negatives, false positives and false negatives, respectively; *N* is the total number of locative proteins and *k* is the class number.

## Results and discussion

We first use dipeptide information with the blank space in ZD98 and CL317 datasets. By the form of “XDY”, a 400-dimension vector was gotten, and then was put into SVM to predict. The predicted results with the change of the parameter *d* are shown in Fig. 2.

From Fig. 2, Number of spaces *d* don’t have very big effect on the prediction classification. In CL317 dataset, with the increasing of *d*, the prediction result tends to decline. It shows that dipeptide information with the blank space is not very good.

By the extraction algorithm based on Gapped *k*-mer, the dimension of eigenvectors and overall accuracy in different *k* value and *g* value are shown in Tables 3 and 4.

**Table 3:** the prediction results based on Gapped *k*-mer of ZD98 data set

k	Space(g)	Dimension	OA(%)
2	0	36	87.76
2	1	12	84.69
3	0	221	88.78
3	1	108	89.80
3	2	18	85.71
4	0	1071	90.82
4	1	849	91.84
4	2	216	90.82
4	3	24	86.73
5	0	3732	93.88
5	1	5351	92.86
5	2	2127	93.88
5	3	360	89.80
5	4	30	87.76
6	0	8698	92.86
6	1	22,263	92.86
6	2	15,986	93.88
6	3	4260	93.88
6	4	540	91.84
6	5	36	88.78

**Table 4:** the prediction results based on Gapped *k*-mer of CL317 data set

k	Space	Dimension	OA(%)
2	0	36	82.22
2	1	12	71.75
3	0	216	86.23
3	1	108	86.98
3	2	18	75.56
4	0	1234	88.89
4	1	864	88.89
4	2	216	88.89
4	3	24	77.78
5	0	5607	87.94
5	1	6145	90.48
5	2	2158	88.89
5	3	360	89.84
5	4	30	81.59
6	0	17,637	90.16
6	1	33,470	90.48
6	2	18,424	90.48
6	3	4316	89.84
6	4	540	90.16
6	5	36	82.22

From Tables 3 and 4, we can see, by the two dimension reduction measures, the dimension of feature information extraction of the *k*-mer polypeptide Gapped has been reduced a lot, Note that a blank line in the table 0 is the results of *k*-mer feature method, the result show that Gapped *k*-mer are better than k-mer. Moreover, the number of space is better not too lager, best one or two spaces, which can be considered as in the protein evolution, mutations occur only in a few location of protein sequences, while the majority of other position without mutations, by matching with a few space, we can match the similarity of two protein sequences better, as above, “BBB” and “BBW”, they are regarded as the same type “BB_” in statistics with a space of 3-peptide information. Namely the space can matching any character, so in the process of practice, use the “BB_” to regular matching the original sequence, statistic the frequency. Conversely, “BBW” can belong to “BB_”, but to “_BW” and “B_W”, namely a variety of types of Gapped *k*-mer can matching to a *k*-peptide in the sequence, compared to the precise *k*-mer matching, the fuzzy matching can extract more similar information.

**Fig. 3:**
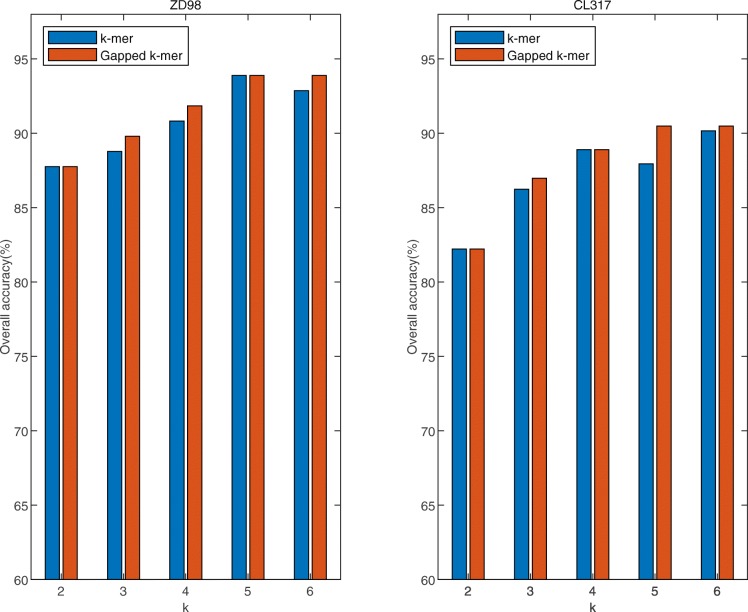
The highest overall accuracy of two data sets correspond to different k values

Figure 3 show the highest Overall accuracy of *k*-mer and Gapped *k*-mer on two data sets, respectively. The results show that with the k value increase, the accuracy of the two methods is generally improved, when extracting the same *k*-peptide information, the accuracy of Gapped *k*-mer is higher than *k*-mer in whole. The accuracy of Gapped *k*-mer reached a maximum value both in ZD98 and CL317. But accurate did not improve when *k* = 6, that meaning unable to extract more information at this time. So the effect is best when *k* = 5 in using the Gapped *k*-mer method to extract feature information.

To illustrate the effectiveness of Gapped *k*-mer method, we compare it with other approaches that have been reported. In recent years, mostly through a variety of characteristics of method of information fusion in the protein subcellular location prediction research. In order to facilitate comparison, we also incorporate other information. At present, many scholars use GO information or GO information and it features information fusion used for protein subcellular localization prediction. Here, we fusing GO information with Gapped *k*-mer (*k* = 5, *g* = 1), SVM as classifier, compare with other method, the result in Table 5 show that the accuracy of our fusion method is relatively high. The overall accuracy increased by 2.5%.

**Table 5:** Comparison of the results of Gneg1456 data sets

Methods	Overall
iLoc-Gneg by Xiao et al. [1]	91.4%
Li and Yu [38]	93.2%
Gneg-mPLoc by Shen and Chou [39]	85.7%
The proposed method	93.3%

## Conclusions

In this paper, we proposed a new method to predict protein subcellular localization based on Gapped *k*-mer. Extracting reduced *k*-peptide component information with the space *k*-peptide representation. Then multi-tree calculating Gapped *k*-mer was also introduced. The new feature representation are used to construct a model and combined with dimension reduction to make the subcellular localization prediction of proteins. Meanwhile, we also discussed the effects of different parameters on the experimental results. The influence of parameters *k* and *g* on the experiment are discussed. Prediction accuracy got the highest when *k* = 5, *g* = 1. Compared to *k*-mer, our method not only reduces the dimension, but also improves the prediction precision, as shown in Table 5.

Li and Yu [38] applied three feature representation: evolutionary information from PSI-blast profile, physiochemical properties and structural features by PROFEAT, gene ontology formulation. Their prediction accuracy for the same dataset adopted in this paper is much higher than other methods, respectively with Jackknife test. We used Gapped *k*-mer and GO information, no matter overall accuracy or absolute true overall accuracy, the achieved results by the proposed method are much higher than Li and Yu.

## Data Availability

The datasets used and/or analysed during the current study are available from the corresponding author on reasonable request.

## Abbreviations

FKNN: fuzzy K-nearest neighbor
MCC: Matthew’s correlation coefficient
OA: overall accuracy
OVR: one-versus-rest
PseAAC: pseudo amino acid composition
RBF: radial basis function
SVM: support vector machine
